# Supplementary material for: Neural representation of abstract task structure during generalization
Source: eLife. 2021 Mar 17;10:e63226. doi: 10.7554/eLife.63226 (PMC8016482; doi:10.7554/eLife.63226)
Supplement: Supplementary file 1. — All reported clusters were significant at the p<0.05, corrected for multiple comparisons after peak thresholding at p<0.001 and permutation-based cluster correction. The critical cluster extent threshold for each contrast is given by the value of k. [file elife-63226-supp1.docx]

**Supplementary Table 1. Activations passing permutation-based cluster correction for whole-brain representational similarity analysis**

| Region (AAL2) | MNI Coordinates | | | Number of voxels | Peak *t*-value |
| --- | --- | --- | --- | --- | --- |
|  | x | y | z |  |  |
| **Latent state**  (k = 760) |  |  |  |  |  |
| Left inferior frontal gyrus, triangularis | -34.5 | 42 | 4.5 | 11206 | 7.49 |
| Left precentral gyrus | -51 | -4.5 | 49.5 |  | 7.25 |
| Left precentral gyrus | -55.5 | 6 | 22.5 |  | 5.80 |
| Right postcentral gyrus | 43.5 | -30 | 42 | 1265 | 7.16 |
| Right inferior parietal gyrus | 58.5 | -45 | 49.5 |  | 4.22 |
| Left middle temporal gyrus | -63 | -13.5 | -10.5 | 12763 | 6.79 |
| Right precuneus | 6 | -72 | 51 |  | 6.53 |
| Left calcarine fissure | -9 | -63 | 18 |  | 6.14 |
| Right insula | 34.5 | 22.5 | 12 | 1807 | 6.11 |
| Right middle frontal gyrus | 40.5 | 55.5 | 9 |  | 5.57 |
| Right middle frontal gyrus | 45 | 30 | 34.5 | 1807 | 3.97 |
| Left inferior parietal gyrus | -49.5 | -37.5 | 45 | 1334 | 6.07 |
|  |  |  |  |  |  |
| **Context**  (k = 726) |  |  |  |  |  |
| Right fusiform gyrus | 31.5 | -48 | -6 | 1386 | 6.04 |
| Left fusiform gyrus | -25.5 | -64.5 | -16.5 | 1102 | 5.30 |
| Left middle occipital gyrus | -22.5 | -63 | 39 | 948 | 5.11 |
|  |  |  |  |  |  |
| **Value**  (k = 760) |  |  |  |  |  |
| Left superior frontal gyrus, dorsolateral | -27 | 58.5 | -1.5 | 2473 | 6.01 |
| Left anterior orbital gyrus | -27 | 42 | -13.5 |  | 5.75 |
| Left middle temporal gyrus | -52.5 | -49.5 | 10.5 | 2140 | 4.85 |
| Left angular gyrus | -45 | -52.5 | 34.5 |  | 4.44 |
| Left middle occipital gyrus | -27 | -63 | 37.5 |  | 4.41 |
| Left superior frontal gyrus, dorsolateral | -15 | 46.5 | 40.5 | 808 | 4.66 |
|  |  |  |  |  |  |
| **Category**  (k = 560) |  |  |  |  |  |
| Vermis 7 | 0 | -76.5 | -19.5 | 183997 | 11.28 |
| Right inferior temporal gyrus | 46.5 | -57 | -13.5 |  | 10.69 |
| Right Cerebelum 4 5 | 25.5 | -33 | -24 |  | 10.48 |
| Left posterior orbital gyrus | -36 | 25.5 | -22.5 | 1962 | 6.07 |
| Left fusiform gyrus | -31.5 | -3 | -40.5 |  | 4.54 |
| Left amygdala | -27 | -1.5 | -18 |  | 4.45 |
| Right supplementary motor area | 15 | -10.5 | 67.5 | 563 | 5.75 |
|  |  |  |  |  |  |
| **Latent State X Value x -1**  (k = 721) |  |  |  |  |  |
| Right inferior occipital gyrus | 48 | -76.5 | -13.5 | 2139 | 6.54 |
|  |  |  |  |  |  |
| **Context X Value**  (k = 681) |  |  |  |  |  |
| Right inferior occipital gyrus | 48 | -78 | -7.5 | 865 | 5.30 |
| Left middle occipital gyrus | -48 | -79.5 | 1.5 | 1015 | 4.83 |
| Left occipital gyrus | 48 | -78 | -7.5 |  | 4.59 |
|  |  |  |  |  |  |
| **Context X Category**  (k = 742) |  |  |  |  |  |
| Left lingual gyrus | -27 | -88.5 | -18 | 8442 | 8.57 |
| Left middle occipital gyrus | -25.5 | -82.5 | 15 |  | 6.63 |
| Right lingual gyrus | 21 | -82.5 | -10.5 |  | 6.57 |
| Right inferior occipital gyrus | 48 | -78 | -7.5 | 1293 | 6.27 |
| Right middle temporal gyrus | 52.5 | -72 | 15 |  | 4.44 |
