# Supplementary material for: Neural representation of abstract task structure during generalization
Source: eLife. 2021 Mar 17;10:e63226. doi: 10.7554/eLife.63226 (PMC8016482; doi:10.7554/eLife.63226)
Supplement: Supplementary file 2. — All reported clusters were significant at the p<0.05, corrected for multiple comparisons after peak thresholding at p<0.001 and permutation-based cluster correction within an explicit mask defining orbitofrontal cortex. The critical cluster extent threshold for each contrast is given by the value of k. [file elife-63226-supp2.docx]

**Supplementary Table 2. Activations passing permutation-based cluster correction for representational similarity analysis constrained to orbitofrontal cortex region of interest**

| Region (AAL2) | MNI Coordinates | | | Number of voxels | Peak *t*-value |
| --- | --- | --- | --- | --- | --- |
|  | x | y | z |  |  |
| **Value**  (k = 57) |  |  |  |  |  |
| Left superior frontal gyrus, dorsolateral | -27 | 58.5 | -1.5 | 996 | 6.01 |
| Left anterior orbital gyrus | -27 | 42 | -13.5 | 996 | 5.75 |
| Left gyrus rectus | -4.5 | 57 | -21 | 57 | 4.32 |
|  |  |  |  |  |  |
| **Latent state**  (k = 44) |  |  |  |  |  |
| Left medial orbital gyrus | -19.5 | 42 | -19.5 | 232 | 6.05 |
| Right middle frontal gyrus | 36 | 58.5 | -3 | 169 | 5.05 |
| Left middle frontal gyrus | -42 | 43.5 | -1.5 | 211 | 4.87 |
