# Supplementary material for: Neural representation of abstract task structure during generalization
Source: eLife. 2021 Mar 17;10:e63226. doi: 10.7554/eLife.63226 (PMC8016482; doi:10.7554/eLife.63226)
Supplement: Supplementary file 3. — All reported clusters were significant at the p<0.05, corrected for multiple comparisons after peak thresholding at p<0.001 and permutation-based cluster correction. The critical cluster extent threshold for each contrast is given by the value of k. [file elife-63226-supp3.docx]

**Supplementary Table 3. Activations passing permutation-based cluster correction for univariate contrast of correct and erroneous responses**

| Region (AAL2) | MNI Coordinates | | | Number of voxels | Peak *t*-value |
| --- | --- | --- | --- | --- | --- |
|  | x | y | z |  |  |
| **Correct > error**  (k = 317) |  |  |  |  |  |
| Right hippocampus | 40.5 | -22 | -17.5 | 576 | 4.56 |
| Left superior temporal gyrus | -61.5 | -26.5 | 5 | 22560 | 6.79 |
| Left medial orbital gyrus | -7.5 | 48.5 | -14.5 | 558 | 4.92 |
| Right middle temporal gyrus | 40.5 | -40 | -8.5 | 349 | 4.70 |
