# Supplementary material for: Neural representation of abstract task structure during generalization
Source: eLife. 2021 Mar 17;10:e63226. doi: 10.7554/eLife.63226 (PMC8016482; doi:10.7554/eLife.63226)
Supplement: Transparent reporting form [file elife-63226-transrepform.docx]

***eLife’s* transparent reporting form**

We encourage authors to provide detailed information *within their submission* to facilitate the interpretation and replication of experiments. Authors can upload supporting documentation to indicate the use of appropriate reporting guidelines for health-related research (see [EQUATOR Network](http://www.equator-network.org/%20)), life science research (see the [BioSharing Information Resource](https://biosharing.org/)), or the [ARRIVE guidelines](http://www.plosbiology.org/article/info:doi/10.1371/journal.pbio.1000412) for reporting work involving animal research. Where applicable, authors should refer to any relevant reporting standards documents in this form.

If you have any questions, please consult our Journal Policies and/or contact us: [editorial@elifesciences.org](mailto:editorial@elifesciences.org).

**Sample-size estimation**

- You should state whether an appropriate sample size was computed when the study was being designed
- You should state the statistical method of sample size computation and any required assumptions
- If no explicit power analysis was used, you should describe how you decided what sample (replicate) size (number) to use

Please outline where this information can be found within the submission (e.g., sections or figure legends), or explain why this information doesn’t apply to your submission:

A description of the basis of our sample size estimate can be found in the Materials and methods section in the second paragraph of the *Participants* sub-section. We also included the same rationale in our pre-registration.

**Replicates**

- You should report how often each experiment was performed
- You should include a definition of biological versus technical replication
- The data obtained should be provided and sufficient information should be provided to indicate the number of independent biological and/or technical replicates
- If you encountered any outliers, you should describe how these were handled
- Criteria for exclusion/inclusion of data should be clearly stated
- High-throughput sequence data should be uploaded before submission, with a private link for reviewers provided (these are available from both GEO and ArrayExpress)

Please outline where this information can be found within the submission (e.g., sections or figure legends), or explain why this information doesn’t apply to your submission:

Detailed information about the number of participants recruited and included in analyses is provided in the first paragraph of the Participants sub-section within the Materials and methods section (i.e. biological replicates).

Information about the number of observations (trials) for each participant (i.e. technical replicates) are provided in the description of the *Experimental procedures* in the Materials and methods section, and are also summarized in Figure 1 – figure supplement 1.

Inclusion and exclusion criteria for participants and data are provided in the *Participants* section in the Materials and methods. These criteria are also described in our preregistration.

Procedures for dealing with outlier volumes in fMRI analysis are provided in the *fMRI preprocessing and analysis* sub-section of the Materials and methods.

**Statistical reporting**

- Statistical analysis methods should be described and justified
- Raw data should be presented in figures whenever informative to do so (typically when N per group is less than 10)
- For each experiment, you should identify the statistical tests used, exact values of N, definitions of center, methods of multiple test correction, and dispersion and precision measures (e.g., mean, median, SD, SEM, confidence intervals; and, for the major substantive results, a measure of effect size (e.g., Pearson's r, Cohen's d)
- Report exact p-values wherever possible alongside the summary statistics and 95% confidence intervals. These should be reported for all key questions and not only when the p-value is less than 0.05.

Please outline where this information can be found within the submission (e.g., sections or figure legends), or explain why this information doesn’t apply to your submission:

Descriptions of each statistical test for behavioral analyses and model-based analyses accompany the textual description of each analysis in the Results section. Figure captions also contain information about symbols signifying p-values for specific comparisons shown in these figures.

Detailed information about statistical analyses of fMRI data are provided in the ‘searchlight analyses’ and ‘region of interest analyses’ sub-sections of the Methods. Specific cluster size values (k) for permutation-derived statistical thresholds are provided accompanying relevant results in figures and in accompanying supplementary tables.

(For large datasets, or papers with a very large number of statistical tests, you may upload a single table file with tests, Ns, etc., with reference to sections in the manuscript.)

**Group allocation**

- Indicate how samples were allocated into experimental groups (in the case of clinical studies, please specify allocation to treatment method); if randomization was used, please also state if restricted randomization was applied
- Indicate if masking was used during group allocation, data collection and/or data analysis

Please outline where this information can be found within the submission (e.g., sections or figure legends), or explain why this information doesn’t apply to your submission:

Information on how participants were sorted into fMRI and behavioral groups can be found in the ‘experimental design’ and ‘participants’ sub-sections of the Methods.

**Additional data files (“source data”)**

- We encourage you to upload relevant additional data files, such as numerical data that are represented as a graph in a figure, or as a summary table
- Where provided, these should be in the most useful format, and they can be uploaded as “Source data” files linked to a main figure or table
- Include model definition files including the full list of parameters used
- Include code used for data analysis (e.g., R, MatLab)
- Avoid stating that data files are “available upon request”

Please indicate the figures or tables for which source data files have been provided:

Raw behavioral data for all participants in the fMRI and behavioral groups is available for download from the OSF data repository for this project (Figures 2-3). All unthresholded t-statistical maps and raw data for ROI-based analyses, as well as *.nii files for our original ROIs are also all available in this repository (Figures 5-7; Supplementary Tables 1-3). Negative log likelihoods for all reaction time models for individual subjects summarized in Table 1 are provided in Table 1-source data 1.
